# Near-Complete Proviral Genome Sequence of Reticuloendotheliosis Virus Isolated from an Attwater’s Prairie Chicken (*Tympanuchus cupido attwateri*)

**DOI:** 10.1128/mra.00173-22

**Published:** 2022-03-23

**Authors:** Bianca Willis, Brittany Stewart, Faith Cox, Tiffany Lujan, Holly Haefele, Camila Romano, Jeff Brady, Dustin Edwards

**Affiliations:** a Department of Biological Sciences, Tarleton State University, Stephenville, Texas, USA; b Fossil Rim Wildlife Center, Glen Rose, Texas, USA; c Instituto de Medicina Tropical de São Paulo e Hospital das Clínicas da Faculdade de Medicina, Universidade de São Paulo, São Paulo, Brazil; d Texas A&M AgriLife Research, Stephenville, Texas, USA; DOE Joint Genome Institute

## Abstract

We report the near-complete proviral genome sequence of a reticuloendotheliosis virus isolated and propagated from an endangered Attwater’s prairie chicken (*Tympanuchus cupido attwateri*) during a 2016–2017 outbreak at a captive breeding facility.

## ANNOUNCEMENT

Reticuloendotheliosis virus (REV), a member of the *Retroviridae* family and *Gammaretrovirus* genus, is an immunosuppressive avian virus that infects B lymphocytes and has been detected in Anseriformes, Galliformes, and Passeriformes (1). In 2016 to 2017, the virus was the leading cause of death among adult endangered Attwater’s prairie chickens (APCs) (*Tympanuchus cupido attwateri*) at the Fossil Rim Wildlife Center (FRWC) captive breeding facility in Glen Rose, Texas (2). REV was previously isolated and sequenced from APC-566 (GenBank accession number DQ387450) during an outbreak at the facility 10 years earlier (3). We performed next-generation sequencing of a 2017 isolate for comparison between outbreaks. Blood from APC-2794, diagnosed as REV positive by quantitative PCR (qPCR) at the Texas A&M Veterinary Medical Diagnostic Laboratory (College Station, TX), was drawn into EDTA-coated tubes by FRWC veterinarians in January 2017 as part of normal practices. Blood was received and diluted 1:3 in phosphate-buffered saline (PBS), and mononuclear cells were isolated by Ficoll-Paque density gradient centrifugation. The buffy coat layer was aspirated, washed in PBS, and cocultivated for 5 days with chicken embryonic fibroblast DF-1 cells (CRL-12203; ATCC) with 5% CO_2_ at 39°C in Dulbecco's modified Eagle medium (11965-092; Gibco) supplemented with 10% fetal bovine serum (20082-147; Gibco) (4). After incubation, supernatant containing released REV was collected, passed through 0.22-μm filters, and placed on fresh DF-1 cells for 5 days, which was repeated for a total of three passages. After the final passage, cells were washed with PBS, pelleted by centrifugation, resuspended in cell culture freezing medium (12646-010; Gibco), aliquoted, and stored in liquid nitrogen. Supernatant containing REV was stored at −80°C, and a frozen aliquot was submitted to Charles River Avian Services (Wilmington, MA) and determined to contain a 50% tissue culture infective dose (TCID_50_) of 6.8 × 10^5^ virions/mL.

Genomic DNA containing provirus was extracted by incubating approximately 1 × 10^6^ REV-infected DF-1 cells with 75 μL HotSHOT alkaline lysis reagent at 95°C for 15 min and was cooled on ice for 5 min before the addition of 75 μL of HotSHOT neutralization solution (5). A genomic sequencing library was prepared at Texas A&M AgriLife Research (Stephenville, TX) using KAPA HiFi HotStart ReadyMix (07958927001; Roche) and primers with Illumina adapters to amplify three overlapping sections, i.e., nucleotide positions 1 to 5075, 1500 to 7295, and 7052 to 8295 (3). Resolved PCR products were excised, pooled, tagmented using the Nextera XT DNA library preparation kit (FC-131-1096; Illumina), and sequenced with an Illumina MiSeq instrument at Texas A&M Genomics and Bioinformatics Services (College Station, TX). Sequencing produced a total of 35,372 paired-end reads of 300-base read length. Primer sequences and low-quality and short (<30-bp) reads were trimmed. A consensus sequence was assembled from sequence reads of two libraries for high coverage, prepared from the same DNA extraction, and a reference (GenBank accession number DQ387450) using the CLC Genomics Workbench v6 assembly to reference tool (mismatch cost=1, indels cost=2, minimum fraction of similarity between reads and reference=90%). A total of 92.4% of the reads were mapped (average coverage, 1,948×). Genome annotation was formatted with SeqBuilder Pro v17.2.1. All tools were run with default parameters unless indicated otherwise. The assembled sequence is 8,246 bp, representing 99.5% of the genome, and has a G+C content of 52.3%. Nucleotide alignment of entire genome sequences with BLASTn (https://blast.ncbi.nlm.nih.gov) (6) showed that the virus is well conserved, with 99.9% nucleotide identity to strain APC-566 with a single nucleotide polymorphism at position 6653 (Table 1). APC-2794 shared 100% nucleotide identity with strain 104865 (GenBank accession number KJ756349), which was isolated from a domestic backyard turkey.

**TABLE 1:** Reference REV isolates used for strain comparisons

GenBank accession no.	Host	Country	Year of isolation	Identity (%)^*a*^
OL857288	APC	USA	2017	
KJ756349	Turkey	USA	2014	100.00
DQ387450	APC	USA	2005	99.99
MF631845	Chicken	Thailand	2013	99.93
GQ415646	Chicken	China	2009	99.89
FJ439119	Goose	Taiwan	2006	99.89
JQ804915	Duck	China	2011	99.84

a Percent identity is based on nucleotide alignments obtained from BLASTn.

### Data availability.

This sequence was deposited under GenBank accession number OL857288. Raw reads are available under SRA accession numbers SRX11820216 and SRX13461851.
